# Molecular diagnosis and novel genes and phenotypes in a pediatric thoracic insufficiency cohort

**DOI:** 10.1038/s41598-023-27641-0

**Published:** 2023-01-18

**Authors:** Alanna Strong, Meckenzie Behr, Carina Lott, Abigail J. Clark, Frank Mentch, Renata Pellegrino Da Silva, Danielle R. Rux, Robert Campbell, Cara Skraban, Xiang Wang, Jason B. Anari, Benjamin Sinder, Patrick J. Cahill, Patrick Sleiman, Hakon Hakonarson

**Affiliations:** 1 Division of Human Genetics, Children’s Hospital of Philadelphia, Philadelphia, PA USA; 2 The Center for Applied Genomics, Children’s Hospital of Philadelphia, Philadelphia, PA USA; 3 Division of Orthopedics, Children’s Hospital of Philadelphia, Philadelphia, PA USA; 4 Department of Pediatrics, Perelman School of Medicine, Children’s Hospital of Philadelphia, University of Pennsylvania, 3615 Civic Center Blvd, Philadelphia, PA 19104 USA; 5 Endowed Chair in Genomic Research, Division of Pulmonary Medicine, The Joseph Stokes, Jr. Research Institute, Children’s Hospital of Philadelphia, Philadelphia, PA USA

**Keywords:** Genetics, Physiology

## Abstract

Thoracic insufficiency syndromes are a genetically and phenotypically heterogeneous group of disorders characterized by congenital abnormalities or progressive deformation of the chest wall and/or vertebrae that result in restrictive lung disease and compromised respiratory capacity. We performed whole exome sequencing on a cohort of 42 children with thoracic insufficiency to elucidate the underlying molecular etiologies of syndromic and non-syndromic thoracic insufficiency and predict extra-skeletal manifestations and disease progression. Molecular diagnosis was established in 24/42 probands (57%), with 18/24 (75%) probands having definitive diagnoses as defined by laboratory and clinical criteria and 6/24 (25%) probands having strong candidate genes. Gene identified in cohort patients most commonly encoded components of the primary cilium, connective tissue, and extracellular matrix. A novel association between *KIF7* and *USP9X* variants and thoracic insufficiency was identified. We report and expand the genetic and phenotypic spectrum of a cohort of children with thoracic insufficiency, reinforce the prevalence of extra-skeletal manifestations in thoracic insufficiency syndromes, and expand the phenotype of *KIF7* and *USP9X*-related disease to include thoracic insufficiency.

## Introduction

Thoracic insufficiency syndromes (TIS) are a phenotypically and genetically heterogeneous group of disorders characterized by respiratory compromise from thoracic and vertebral malformations or deformations^1^. These conditions carry high morbidity and mortality from pulmonary compromise. Treatment is aimed at expanding the thoracic cavity to optimize lung growth. The vertical expandable prosthetic titanium rib (VEPTR) device has evolved as an important tool to expand the thoracic cavity and promote pulmonary function, weight gain, growth, and quality of life^2^. Despite the advances in medical and surgical care for children with TIS, the phenotypic and genetic landscape of these disorders remain incompletely understood.

Many TIS occur secondary to underlying genetic disease, which interfere with proper skeletogenesis, skeletal maintenance and growth, or skeletal support^3,4^. Symptomatology can be limited to the skeleton (non-syndromic) or can involve multiple organ systems (syndromic). The most common syndromic skeletal dysplasia associated with thoracic insufficiency are the short-rib thoracic dysplasia syndromes, characterized by short ribs and thoracic insufficiency often accompanied by polydactyly, dwarfism, kidney and liver disease, congenital heart disease and retinopathy^5^. Causal genes include *DYNC2H1, IFT172, IFT180, TTC21B, WDR19,* and *WDR34*, which encode components of the primary cilium, a mechanosensory organelle that helps cells sense and respond to their environment, facilitating skeletal patterning by an unknown mechanism^6^.

Thoracic insufficiency can also be caused by pathogenic variants in genes encoding components of connective tissue. Specifically, *COL2A1* encodes a component of type II collagen, and heterozygous variants in this gene are associated with a spectrum of skeletal dysplasias that cause thoracic insufficiency through rib hypoplasia due to impaired skeletogenesis^7^. Neuromuscular disease can cause thoracic insufficiency through inadequate support of the spine and thoracic cavity. This is commonly seen in muscular dystrophy syndromes such as Duchenne muscular dystrophy caused by hemizygous pathogenic variants in *DMD*^8^.

Causal genetic variants for TIS most commonly localize within the protein-coding region of the gene and cause disease either through a loss-of-function (insufficient protein made) or gain-of-function (abnormal protein made that interferes with the function of the normal protein) mechanism. Regardless of underlying cause, molecular diagnosis is critical in TIS and may help determine recurrence risk, associated comorbidities, disease severity, and risk of progression of restrictive lung disease^4,9^. Advances in next-generation sequencing have improved the molecular diagnosis rate for skeletal malformation syndromes. Molecular etiology is identified in 50–90% of individuals with skeletal dysplasia; however, molecular diagnosis is rarer in individuals with scoliosis without skeletal dysplasia or in individuals with non-syndromic scoliosis due to our incomplete understanding of the genetics of skeletal malformations^10–18^. Additionally, despite molecular diagnosis, there remains uncertainty about disease course due to the rarity of these conditions and lack of long-term natural history studies delineating the full phenotypic spectrum.

In this study, we have assembled a cohort of 42 individuals who presented to the Orthopedic clinic at The Children’s Hospital of Philadelphia (CHOP) for evaluation and management of thoracic insufficiency and performed comprehensive phenotyping analysis and whole exome sequencing to better elucidate the genetic and phenotypic landscape of this disorder.

## Methods

### Patient recruitment

Patients referred to Orthopedics at The Children’s Hospital of Philadelphia (CHOP) for evaluation and management of thoracic insufficiency were eligible for study participation. Participants were recruited from the inpatient and outpatient settings. Inpatients were recruited from the inpatient service at the Main Campus hospital in Philadelphia. Outpatients were recruited from the Orthopedic clinics at CHOP Main Campus and from regional CHOP outpatient sites. All recruitment took place between the years 2015–2020. A total of 42 families consented for enrollment. A blood sample was obtained upon enrollment and used for DNA extraction, microarray analysis, and next generation sequencing. Parental samples were obtained when possible for segregation analysis and to phase variants. This study was approved by the Children’s Hospital of Philadelphia institutional review board (Protocol # 16-013278) and all studies were performed in accordance with the Declaration of Helsinki. Informed consent was obtained from all participants and/or their legal guardians. Specifically, all 42 participants agreed to participate in this study and signed appropriate consent forms. For children under 18 years parental consent was obtained for participation and children’s assent was obtained when possible for children 7 years or older.

### Phenotyping

Skeletal phenotyping was done by Orthopedic physicians at CHOP. Extra-skeletal phenotyping was performed by other subspecialists that evaluated the patients. Clinical care was not influenced by study participation. All charts were reviewed by a Board-certified clinical geneticist for phenotyping analysis. Chemistry laboratories, imaging studies and patient photographs were reviewed when available.

### Exome sequencing

Exome sequencing was performed by the Center for Applied Genomics (CAG) at CHOP using the Twist Human Core Exome Capture Kit (TWIST Bioscience) and Illumina NovaSeq 6000. Approximately 33 million 100 base pair paired-end reads were generated with median insert size of 250 base pairs. Data were quality controlled and analyzed using a custom-built pipeline that incorporates BWA-mem v0.7.12 for alignment, Picard v1.97 for PCR duplication removal, and GATK v2.6.5 for variant calling. On average, 97.4% of each patient’s exome was sequenced at a depth of at least 20×. Variant annotation, filtration and prioritization was performed with GDCross, a variant annotation and prioritization platform developed within the CAG and validated for clinical use. Variants with ≥ 5× coverage were initially filtered at 0.5% gnomAD mean allele frequency and annotated with a combination of Variant Effect Predictor, HGMD, ClinVar, dbSNP, OMIM, HPO, PolyPhen-2 and SIFT, and a custom-built splice-site annotator^19,20^. Variants were priority ranked using a weighted combination of (a) overlap with HPO terms, (b) patient and family genotypes, (c) predicted functional impact, (d) inheritance modeling, and (e) presence in mutation databases such as HGMD and ClinVar. Variants identified computationally were reviewed by a laboratory geneticist for validation of pathogenicity (Polyphen and SIFT predictions and CADD score^21,22^) and by a clinical geneticist for phenotype match. Sanger validation was performed for all reported variants. Parental testing for variants identified in probands was done via Sanger sequencing.

### Editorial policies and ethical considerations

This study was approved by the Children’s Hospital of Philadelphia institutional review board (Protocol # 16-013278). All studies were performed in accordance with the Declaration of Helsinki. Informed consent was obtained from all participants and/or their legal guardians. Specifically, all members of the family agreed to participate in this study and signed appropriate consent forms.

## Results

### Cohort characteristics

A total of 42 patients were enrolled into CAG for microarray and exome sequencing. Patients were recruited from both the outpatient and inpatient settings. Age at enrollment was 0–11.6 years. There was a slight predominance of females (24/42; 57%) compared to males (18/42; 43%) in the study. Self-assigned races included White (26/42; 62%), Black (8/42; 19%), Asian (4/42; 9.5%), Hispanic White (2/42; 4.8%), and Hispanic Black (1/42; 2.4%). One patient did not specify race (Tables 1, 2, Supplemental Table 1). Representative chest/spine films for all probands are presented in Supplemental Fig. 1.

**Table 1:** Patient demographics and clinical information.

Category	Number
Sex	Female: 24/42 (57%)
	Male: 18/42 (43%)
Age	At enrollment: 0–11.6 years
	Current: 2–18.5 years
Race	White: 26/42 (62%)
	Black: 8/42 (19%)
	Asian: 4/42 (9.5%)
	Hispanic White: 2/42 (4.8%)
	Hispanic Black: 1/42 (2.4%)
	Other: 1/42 (2.4%)
Skeletal diagnoses	Syndromic scoliosis: 20/42 (48%)
	Short-rib thoracic dysplasia 16/42 (41%)
	Isolated scoliosis: 6/42 (14%)
Extra-skeletal manifestations	Developmental delay: 10/42 (24%)
	Endocrinopathies: 8/42 (19%)
	Kidney abnormalities: 7/42 (17%)
	Airway abnormalities: 6/42 (14%)
	Brain abnormalities: 6/42 (14%)
	Cardiac abnormalities: 6/42 (14%)
	Hematological abnormalities: 6/42 (14%)
	Ophthalmological abnormalities: 6/42 (14%)
	Failure to thrive: 5/42 (12%)
	Psychiatric diagnoses: 5/42 (12%)
	Urogenital abnormalities: 4/42 (9.5%)
	Liver abnormalities: 3/42 (7%)
	Recurrent infections: 3/42 (7%)
	Dermatological abnormalities: 2/42 (5%)
Spirometry	% Predicted FVC: 18–97% (n = 17)
	% Predicted FEV1: 14–100% (n = 17)
	% Predicted FEV1/FVC: 44–111% (n = 17)
	% Predicted FEF25-70: 8–123% (n = 17)
	% Predicted PEFR: 30–74% (n = 12)
Plethysmography	% Predicted TLC: 58–107% (n = 11)
	% Predicted VC: 12–79% (n = 8)
	% Predicted FRC: 49–97% (n = 8)
	% Predicted RV: 15–132% (n = 11)
	% Predicted RV/TLC ratio: 35–206% (n = 10)

*FEF* forced expiratory flow; *FEV1* forced expiratory volume, *FRC* functional residual capacity, *FVC* forced vital capacity, *PEFR* peak expiratory flow rate, *RV* residual volume, *TLC* total lung capacity, *VC* vital capacity.

**Table 2:** Cohort phenotypes and genetic variants.

Subject ID	Sex	Race	Key phenotypes	Clinical diagnosis	Spirometry/plethysmography	Identified gene	Variant classification
Subject 1	F	White	Short ribs, hepatomegaly, increased kidney cortical echogenicity	Short-rib thoracic dysplasia 3 with or without polydactyly (OMIM #613091)	% Predicted FVC: 0.44 % Predicted FEV1: 0.46 % Predicted FEV1/FVC: 1.04 % Predicted FEF25-75: 60 % Predicted TLC: 0.58 % Predicted RV: 0.96 % Predicted RV/TLC: 1.59	*DYNC2H1*	Positive
Subject 11	M	White	Short ribs, anemia, bilateral echogenic kidneys	Short-rib thoracic dysplasia 3 with or without polydactyly (OMIM #613091)	–	*DYNC2H1*	Positive
Subject 14	M	White	Short ribs, developmental delay	Short-rib thoracic dysplasia 3 with or without polydactyly (OMIM #613091)	% Predicted FVC: 0.81 % Predicted FEV1: 0.85 % Predicted FEV1/FVC: 1.06 % Predicted FEF25–75: 1.23 % Predicted TLC: 0.8 % Predicted VC: 0.79 % Predicted RV: 0.83 % Predicted RV/TLC: 1.02	*DYNC2H1*	Positive
Subject 21	M	White	Short ribs, autism, chronic kidney disease	Short-rib thoracic dysplasia 3 with or without polydactyly (OMIM #613091)	% Predicted FVC: 0.19 % Predicted FEV1: 0.15 % Predicted FEV1/FVC: 0.81 % Predicted FEF25–75: 0.08 % Predicted PEFR: 0.31 % Predicted TLC: 0.64 % Predicted VC: 0.33 % Predicted FRC: 0.8 % Predicted RV: 1.32 % Predicted RV/TLC: 2.06	*DYNC2H1*	Positive
Subject 23	F	White	Short ribs, hydrocephalus, hydronephrosis, coarse liver echotexture, liver cyst	Short-rib thoracic dysplasia 3 with or without polydactyly (OMIM #613091)	–	*DYNC2H1*	Positive
Subject 29	F	Black	Short ribs, ventriculomegaly	Short-rib thoracic dysplasia 3 with or without polydactyly (OMIM #613091)	–	*DYNC2H1*	Positive
Subject 39	M	White	Short ribs, elevated aminotransferases and GGT	Short-rib thoracic dysplasia 3 with or without polydactyly (OMIM #613091)	–	*DYNC2H1*	Positive
Subject 37	F	White	Thoracolumbar scoliosis, myopia, astigmatism	Congenital Contractural Arachnodactyly (OMIM #121050)	–	*FBN2*	Strong candidate
Subject 2	F	Hispanic White	Craniosynostosis, subglottic stenosis, macrocephaly, hydrocephalus, global developmental delay, thoracic scoliosis	Pfeiffer Syndrome (OMIM #101600)	–	*FGFR2*	Positive
Subject 4	F	White	Thoracic scoliosis	Larsen syndrome (OMIM #150250)	–	*FLNB*	Strong candidate
Subject 30	M	White	Tethered cord, hip dysplasia, vertical talus, scoliosis	Larsen syndrome (OMIM #150250)	% Predicted FVC: 0.77 % Predicted FEV1: 0.84 % Predicted FEV1/FVC: 1.09 % Predicted FEF25-75: 0.86 % Predicted PEFR: 0.71	*FLNB*	Positive
Subject 36	F	Asian	Bilateral talipes equinovarus, bilateral elbow dislocation, left femoral head dislocation, joint contractures	Larsen syndrome (OMIM #150250)	% Predicted FVC: 0.89 % Predicted FEV1: 0.844 % Predicted FEV1/FVC: 0.99 % Predicted FEF25-75: 1.19 % Predicted PEFR: 1.1	*FLNB*	Positive
Subject 16	M	White	Thoracic scoliosis, global developmental delay, cryptorchidism, chordeic penis	X-linked syndromic mental retardation, turner type (OMIM #309590)	–	*HUWE1*	Positive
Subject 38	M	White	Short ribs, retinopathy, bilateral increased kidney echogenicity, decreased corticomedullary differentiation, kidney dysplasia, cortical cyst, dialysis dependence, hydrocephalus	Short-rib thoracic dysplasia 9 with or without polydactyly (OMIM #266920)	–	*IFT140*	Positive
Subject 27	F	Black	Short ribs, hypertension, chronic kidney disease, coarse liver, cerebellar volume loss	Joubert syndrome/acrocallosal syndrome (OMIM #200990)	–	*KIF7*	Strong candidate
Subject 34	F	Asian	Thoracic scoliosis, rib fusions, vertebral fusions and segmentation defects	Spondylocostal dysostosis 2 (OMIM #608681)	% Predicted FVC: 0.52 % Predicted FEV1: 0.66 % Predicted FEF25–75: 2.12 % Predicted TLC: 0.6 % Predicted FRC: 0.53 % Predicted RV: 0.4 % Predicted RV/TLC: 0.75	*MESP2*	Positive
Subject 17	F	Black	Thoracolumbar scoliosis, myopia, pes planus	Freeman–Sheldon syndrome (OMIM #193700)	% Predicted FVC: 0.66 % Predicted FEV1: 0.63 % Predicted FEV1/FVC: 0.96 % Predicted FEF25–75: 0.83 % Predicted PEFR: 0.69 % Predicted TLC: 0.68 % Predicted VC: 0.7 % Predicted FRC: 0.67 % Predicted RV: 0.63 % Predicted RV/TLC: 0.93	*MYH3*	Strong candidate
Subject 15	F	Black	Short ribs, retinopathy, lumbar scoliosis, short stature, chronic kidney disease	Short-rib thoracic dysplasia 6 with or without polydactyly (OMIM #263520)	% Predicted FVC: 0.4 % Predicted FEV1: 0.28 % Predicted FEV1/FVC: 0.72 % Predicted FEF25–75: 0.11 % Predicted PEFR: 0.31	*NEK1*	Positive
Subject 19	M	White	Thoracolumbar scoliosis, developmental delay, autism	Sotos syndrome (OMIM #117550)	–	*NSD1*	Positive
Subject 10	M	Other	Abnormal thoracic cavity, lung hypoplasia, pancreatic insufficiency, failure to thrive, speech delay	Shwachman-Diamond syndrome (OMIM #260400)	–	*SBDS*	Positive
Subject 26	M	White	Hemivertebrae, scoliosis, ambiguous genitalia, lobar holoprosencephaly, global developmental delay, micropenis, imperforate anus, seizures	Intellectual development disorder X-linked 99 (OMIM #300919)	–	*USP9X*	Strong candidate
Subject 9	F	White	Short ribs, micromelia, brachydactyly, hemolytic uremic syndrome, kidney failure, kidney transplant, vision loss	Short-rib thoracic dysplasia 5 with or without polydactyly (OMIM #614376)	% Predicted FVC: 0.97 % Predicted FEV1: 0.67 % Predicted FEV1/FVC: 0.83 % Predicted FEF25–75: 0.24 % Predicted PEFR: 0.71 % Predicted TLC: 0.71 % Predicted VC: 0.77 % Predicted RV: 0.15	*WDR19*	Strong candidate
Subject 7	M	Black Hispanic	Short ribs, failure to thrive, global developmental delay	Short-rib thoracic dysplasia 11 with or without polydactyly (OMIM #613363)	–	*WDR34*	Positive
Subject 41	F	Asian	Short ribs, thoracolumbar kyphosis	Short-rib thoracic dysplasia 11 with or without polydactyly (OMIM #613363)	% Predicted FVC: 0.36 % Predicted FEV1: 0.32 % Predicted FEF25–75: 0.2 % Predicted TLC: 0.68 % Predicted FRC: 0.68 % Predicted RV: 0.52 % Predicted RV/TLC: 135	WDR34	Positive

*FEF* forced expiratory flow, *FEV1* forced expiratory volume, *FRC* functional residual capacity, *FVC* forced vital capacity, *PEFR* peak expiratory flow rate, *RV* residual volume, *TLC* total lung capacity, *VC* vital capacity.

**Figure 1:**
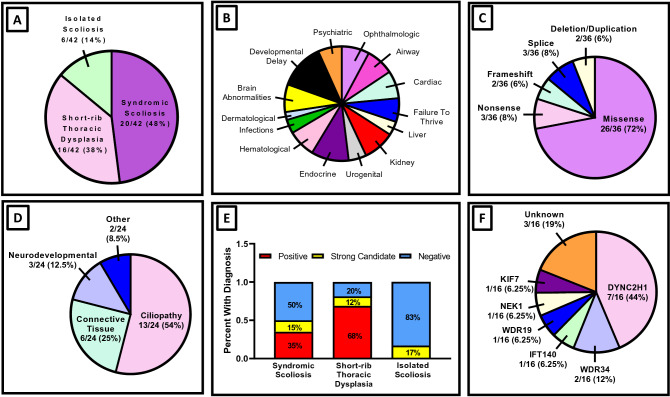
Cohort characteristics. (**A**) Distribution of skeletal diagnoses in cohort patients. (**B**) Common extra-skeletal comorbidities in cohort patients. (**C**) Distribution of variant type identified in cohort patients. (**D**) Category of genes identified in cohort patients. (**E**) Diagnostic success rate for different skeletal phenotypes. (**F**) Genetic landscape of genes identified in cohort patients with short-rib thoracic dysplasia syndromes.

The most common clinical diagnosis was syndromic scoliosis, defined as scoliosis with extra-skeletal manifestations (20/42; 48%), followed by short-rib thoracic dysplasia syndromes (16/42; 38%). Isolated scoliosis was seen in 6/42 patients (14%) (Fig. 1a). Most common extra-skeletal manifestations were developmental delay (10/42; 24%) followed by endocrinopathies (8/42; 19%) and kidney abnormalities (7/42; 17%). Brain malformations, eye abnormalities, airway abnormalities, cardiac abnormalities and hematological abnormalities were seen in 14% of patients (6/42) (Fig. 1b). A subset of patients (17/42) were able to perform pulmonary function testing. Probands had variable degrees of lung disease, ranging from near normal to severe restrictive lung disease. One child had evidence of mixed severe restrictive and obstructive lung disease (Table 1, Supplemental Table 2).

### Variants identified and interpretation

Candidate genes and variants were identified in 57% of probands (24/42). Of these 24 probands, 18/24 (75%) were molecularly diagnosed and 6/24 (25%) had a strong candidate gene identified. A total of 36 variants were identified in 15 different genes. Most variants were missense (26/36; 72%), followed by splice variants (3/36; 8%), nonsense variants (3/36; 8%), frameshift variants (2/36; 6%), and in-frame deletion/duplication (2/36; 6%) (Fig. 1c). Seventeen variants were novel, not observed in gnomAD3.1 or ClinVar (Supplemental Table 3). Ciliopathy-spectrum disease was the most common molecular diagnosis (13/24; 54%), followed by connective tissue and extracellular matrix genes (6/24; 25%), and neurodevelopmental genes (3/24; 12.5%) (Fig. 1d, Tables 2, 3). Individuals with short-rib thoracic dysplasia syndromes were most likely to receive a molecular diagnosis (13/16; 81%), followed by syndromic scoliosis (10/20; 50%). Isolated congenital scoliosis had the lowest diagnostic success rate (1/6; 17%) (Fig. 1e). Of the 16 individuals with a clinical diagnosis of short-rib thoracic dysplasia syndrome, 7 had variants in *DYNC2H1* and 2 had variants in *WDR34*. Variants in *IFT140, WDR19* and *NEK1* were each identified in a single proband. Three probands had negative genetic testing and 1 had variants in *KIF7*, a gene previously not associated with short-rib thoracic dysplasia (Fig. 1f). We additionally identified a maternally-inherited missense variant in *USP9X*, a gene not previously associated with thoracic insufficiency. Clinical details to highlight these phenotypic expansions are detailed below.

**Table 3:** Disease categories and genes identified.

Disease category	Causal gene	Number of patients
Ciliopathy	*DYNC2H1*	7
	*IFT140*	1
	*KIF7*	1 (strong candidate)
	*NEK1*	1
	*WDR19*	1 (strong candidate)
	*WDR34*	2
Neurodevelopmental syndromes	*HUWE1*	1
	*NSD1*	1
	*USP9X*	1 (strong candidate)
Connective tissue disorders and disorders of the extracellular matrix	*FBN2*	1 (strong candidate)
	*FGFR2*	1
	*FLNB*	3 (1/3 strong candidate)
	*MYH3*	1 (strong candidate)
Ribosomopathy	*SBDS*	1
Disorders of cellular signaling	*MESP2*	1

## Case reports

### Case 1 (KIF7)

**Figure 2:**
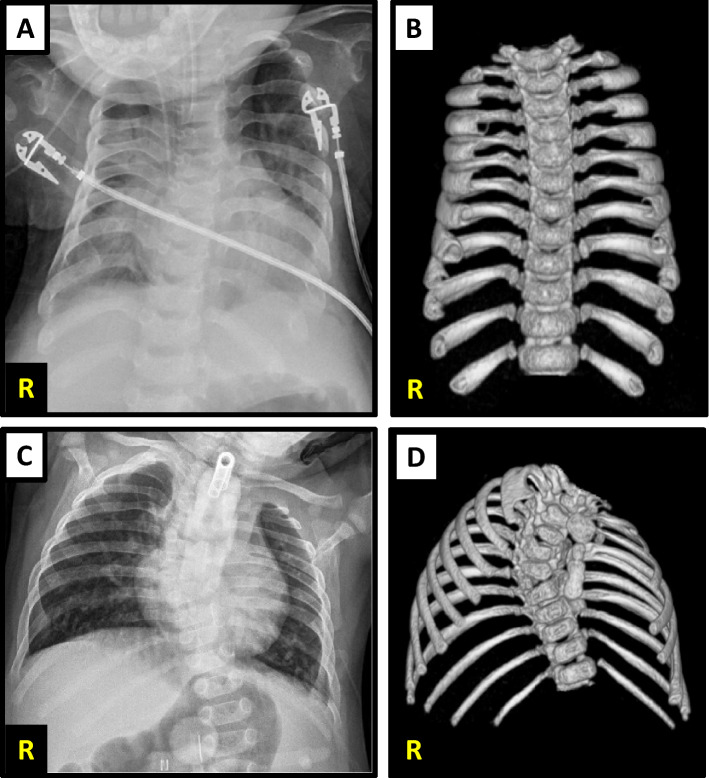
Patient 1 (**A**) Chest X-ray obtained at 2 years of age showing narrow thorax with short ribs and lung hypoplasia. (**B**) 3D reconstruction of the thorax from chest CT images obtained at 2 years of age. Patient 2 (**C**) Spine films obtained at 9 months of age showing thoracic scoliosis, thoracic spine vertebral segmentation defects, left rib fusion defects, and left lung compression. (**D**) 3D reconstruction of the thorax from chest CT images obtained at 1 year of age.

Patient was conceived to a 26-year-old G4P3 → 4 mother. Pregnancy was complicated by deep vein thrombosis and limited prenatal care. Patient was born full-term via vaginal delivery. APGAR scores were 9 at 1 and 5 min. Birth weight was 3.43 kg (53%), length was 49 cm (50%), and head circumference was 36 cm (90%). She was admitted to the NICU for 3 days for respiratory distress and supplemental oxygen requirement, but was ultimately discharged home in room air. At 5 months of age she was diagnosed with torticollis, hypotonia, developmental delay, failure to thrive, and small chest. Skeletal survey showed bilateral metaphyseal flaring of the upper and lower extremity long bones and flaring of the costochondral rib junctions. Abdominal ultrasound showed a small splenule with normal liver and kidney. G-tube was placed at 1 year of age for persistent failure to thrive. She also developed daytime oxygen requirement and nocturnal BiPAP dependence. At 2 years of age she was hospitalized for respiratory failure in the context of pneumonia and was transferred to CHOP for evaluation for VEPTR placement. Chest x-rays and CT showed a bell-shaped chest and shortened, widened and bulbous ribs consistent with a clinical diagnosis of short-rib thoracic dysplasia syndrome (Fig. 2a,b). MRI of the chest and spine demonstrated severe chest wall hypoplasia with hypoplastic lungs, dysmorphic vertebrae with loss of vertebral body height at C7–T3, posterior vertebral body hypoplasia at T10–L3, hypoplastic vertebral body at L5, and a horizontal sacrum. She was incidentally noted to have volume loss in the superior cerebellar hemispheres and a prominent vertebrobasilar system. Brain MRI was not performed. Patient was unable to perform pulmonary function testing due to her young age and disease severity.

At 3 years of age she underwent tracheostomy and VEPTR placement due to persistent respiratory failure. During admission, she was diagnosed with microcytic anemia and hypertension managed with amlodipine and clonidine. Abdominal ultrasound showed increased echogenicity of the renal parenchyma and coarse liver echotexture with echogenic biliary triads. Liver function tests and creatinine levels were normal. Research-based exome sequencing showed 2 *KIF7* variants of uncertain significance: c.2549 G > T; p.Arg850Leu and c.3842 G > T; p.Ser1281Ile.

### Case 2 (USP9X)

Patient is a 7-year-old male born at 34 weeks gestational age to a 29-year-old G6P2 → 3 mother via induced vaginal delivery for maternal pre-eclampsia. Pregnancy was complicated by ultrasounds concerning for agenesis of the corpus callosum confirmed by fetal MRI. Birth weight was 1.843 kg (25%), length was 40 cm (10%), and head circumference was 28 cm (< 5%: 50% for 30 weeks gestation). APGAR scores were 3, 5 and 8 at 1, 5 and 10 min, respectively. He was admitted to the NICU due to multiple congenital anomalies, including ambiguous genitalia with micropenis, lateral facial cleft, preauricular and oral skin tags, left rocker bottom foot, and imperforate anus. The diagnosis of imperforate anus prompted a workup for VACTERL association, which was notable for cor triatriatum, congenital scoliosis, multiple rib and vertebral anomalies, and sacral agenesis. Specifically, chest x-ray showed dextrothoracic scoliosis, multiple vertebral segmentation defects in the thoracic spine, 13 right-sided ribs, and 10 left-sided ribs. CT scan showed fusion of the posterior aspects of the first and second and fourth and fifth ribs on the left, fusion of the first and second ribs on the right, and multiple vertebral segmentation defects in the thoracic spine (Fig. 2c,d). Brain MRI showed agenesis of the corpus callosum and lobar holoprosencephaly.

Patient is currently 7 years of age. Growth parameters are notable for a weight of 17.1 kg (25%) and a height of 110.5 cm (5%). Additional diagnoses include severe global developmental delay, seizures, diabetes insipidus, hearing loss, dysphagia, G-tube dependence, hypothyroidism, and thoracic insufficiency with tracheostomy dependence. VEPTR was ultimately placed at 13 months of age. Prior clinical genetic testing included a chromosomal microarray and a holoprosencephaly gene panel, which were non-diagnostic. Research based exome sequencing was notable for a maternally-inherited variant of uncertain significance in *USP9X* (c.1886 T > C; p.(Leu629Pro)). Of note, there is a history of 3 prior pregnancy losses at 8, 15 and 17 weeks gestation and a vanishing twin. No genetic testing was done on the products of conception.

## Discussion

Thoracic insufficiency syndromes (TIS) have a broad phenotypic and genetic spectrum, and despite advances in next-generation sequencing the diagnostic success rate ranges from 30 to 75% in most studies^10–18^, with particularly low success rates in individuals with isolated scoliosis^23–25^ and particularly high success rates in skeletal dysplasias^26^. We assembled and phenotyped a diverse cohort of children with thoracic insufficiency and identified clear patterns of comorbidities including eye, kidney, brain, cardiac and endocrine disease. Exome sequencing of this cohort identified a molecular etiology in 57% of the participants (24/42), with 18/24 probands having a positive diagnosis identified and 6/24 probands having strong candidate genes identified, consistent with previous cohort studies^10–18^. Identified genes commonly encoded structural and functional components of the primary cilium, bone, and extracellular matrix. Individuals with short-rib thoracic dysplasia syndromes had the highest molecular diagnosis rate (81%), and *DYNC2H1* was the most common causal gene (7/16; 44%). Isolated scoliosis had the lowest diagnosis rate (1/6; 17%). Pulmonary function and response to VEPTR placement appeared independent of causal gene and variant.

Syndromic diagnoses in our cohort included short-rib thoracic dysplasia syndrome, Larsen syndrome (OMIM #150250), Sotos syndrome (OMIM #117550), Pfeiffer syndrome (OMIM #101600), congenital contractural arachnodactyly (OMIM #121050), Intellectual Developmental Disorder X-Linked Type 99 (*USP9X*-related disease, OMIM #300919), X-linked syndromic mental retardation Turner Type (*HUWE1-*related disease, OMIM #309590), Freeman-Sheldon syndrome (OMIM #193700), spondylocostal dysostosis (OMIM #608681), and Shwachman-Diamond syndrome (OMIM #260400). Thoracic insufficiency is a rare but known complication of Shwachman Diamond syndrome^27–30^ and many of the other syndromes represented in our cohort, though has not been previously reported in Pfeiffer syndrome, X-linked syndromic mental retardation Turner Type (*HUWE1-*related intellectual disability), or Intellectual Developmental Disorder X-Linked Type 99 (*USP9X*-related intellectual disability). Of note, there is one prior report of scoliosis in Pfeiffer syndrome, but there was no associated thoracic insufficiency^31^. Severe scoliosis has been reported in the related craniosynostosis Crouzon syndrome^32^, though is rare.

We report expansion of the phenotype of *KIF7* and *USP9X*-related disease to include thoracic insufficiency. *KIF7* encodes kinesin family member 7, a molecular motor that facilitates anterograde transport in the primary cilium and plays a critical role in ciliary transport, structure, and signaling^33^. Biallelic pathogenic *KIF7* variants cause the ciliopathies acrocallosal syndrome (agenesis of the corpus callosum, polydactyly, dysmorphic features) and Joubert syndrome (intellectual disability, hypotonia and cerebellar malformations), but have not been reported in short-rib thoracic dysplasia syndromes^34,35^. We report a patient with biallelic *KIF7* variants and thoracic insufficiency and skeletal dysplasia characteristic of short-rib thoracic dysplasia syndromes with the accompanying features of kidney disease, liver disease, cerebellar volume loss, and developmental delay, which may suggest a novel association between *KIF7* and short-rib thoracic dysplasia syndromes. Of note, biallelic *KIF7* variants can cause Al-Gazali-Bakalinova syndrome, which has a prominent skeletal phenotype including epiphyseal dysplasia, pectus deformities, hip dislocation, genu valgum, and prominent joints, and heterozygous variants in *KIF7* have been reported in idiopathic scoliosis, supporting a role for *KIF7* in skeletal development^36,37^. Our patient’s severe phenotype may reflect severely compromised ciliary function.

*USP9X* maps to the X-chromosome and encodes a deubiquitylase enzyme that prevents the degradation of cellular proteins^38^. *USP9X* plays a critical role in cytoskeleton integrity, chromosome alignment, axonal growth, and neuronal migration^39,40^. Females with heterozygous pathogenic *USP9X* variants can be asymptomatic, but can also have developmental delay, structural brain malformations, dysmorphisms, short stature, choanal atresia, postaxial polydactyly, congenital heart disease, facial clefting, anal atresia, and scoliosis^41,42^. Males with pathogenic *USP9X* variants have severe disease with global developmental delay, brain malformations most commonly involving the ventricles, cerebellum, and corpus callosum, hypotonia, seizures, autism, growth failure, myopia, genitourinary malformations, and scoliosis^43,44^. Though thoracic insufficiency has not been described in Intellectual Developmental Disorder X-Linked Type 99 (*USP9X*-related disease), our patient’s phenotype is consistent with this diagnosis, suggesting that *USP9X* variants can cause thoracic insufficiency. Of note, the protein product of the *USP9X* gene localizes to the primary cilium, and ubiquitination plays a critical role in regulating ciliogenesis and ciliary function^45^. The association between an additional ciliary protein and thoracic insufficiency again highlights the critical role of the primary cilium in thoracic patterning and growth.

Importantly, many of the identified variants in our study are classified as variants of uncertain significance by ACMG criteria, which can be challenging for family planning and clinical application. In silico prediction tools and in vitro studies for genes with biochemical, transcriptional, or functional readouts can help clarify the significance of some of these variants. For causal genes that do not lend themselves to in vitro validation studies, the creation of knockout and transgenic models in established animal models of scoliosis such as zebrafish and mice may help clarify the significance of these variants and establish causality^46–48^. An additional limitation is small cohort size and limited pulmonary function data to more conclusively determine if the causal gene and/or variant can predict pulmonary function over time and response to VEPTR placement.

In conclusion, we present the phenotypic and genetic spectrum of a cohort of 42 individuals with thoracic insufficiency. Our cases suggest that in addition to orthopedic evaluation, all individuals with thoracic insufficiency should have baseline pulmonology, ophthalmology, cardiology, kidney, endocrine and developmental assessments. We additionally reinforce the prevalence of thoracic insufficiency in Shwachman-Diamond syndrome, report the novel finding of pediatric scoliosis and thoracic insufficiency in Pfeiffer syndrome, expand the list of genes associated with short-rib thoracic dysplasia syndromes to include *KIF7*, and report the novel association of *USP9X* and thoracic insufficiency. Altogether, our results highlight the importance of broad-based molecular testing to capture atypical presentations and molecular causes of skeletal malformations.

## Supplementary Information

Supplementary Figure 1.

Supplementary Table 1.

Supplementary Table 2.

Supplementary Table 3.

Supplementary Table 4.

## Data Availability

The datasets used and/or analyzed during the current study available from the corresponding author on reasonable request.
