# Supplementary material for: Molecular diagnosis and novel genes and phenotypes in a pediatric thoracic insufficiency cohort
Source: Sci Rep. 2023 Jan 18;13:991. doi: 10.1038/s41598-023-27641-0 (PMC9849333; doi:10.1038/s41598-023-27641-0)
Supplement: Supplementary file 1 — Supplementary Figure 1. [file 41598_2023_27641_MOESM1_ESM.pptx]

## Slide 1
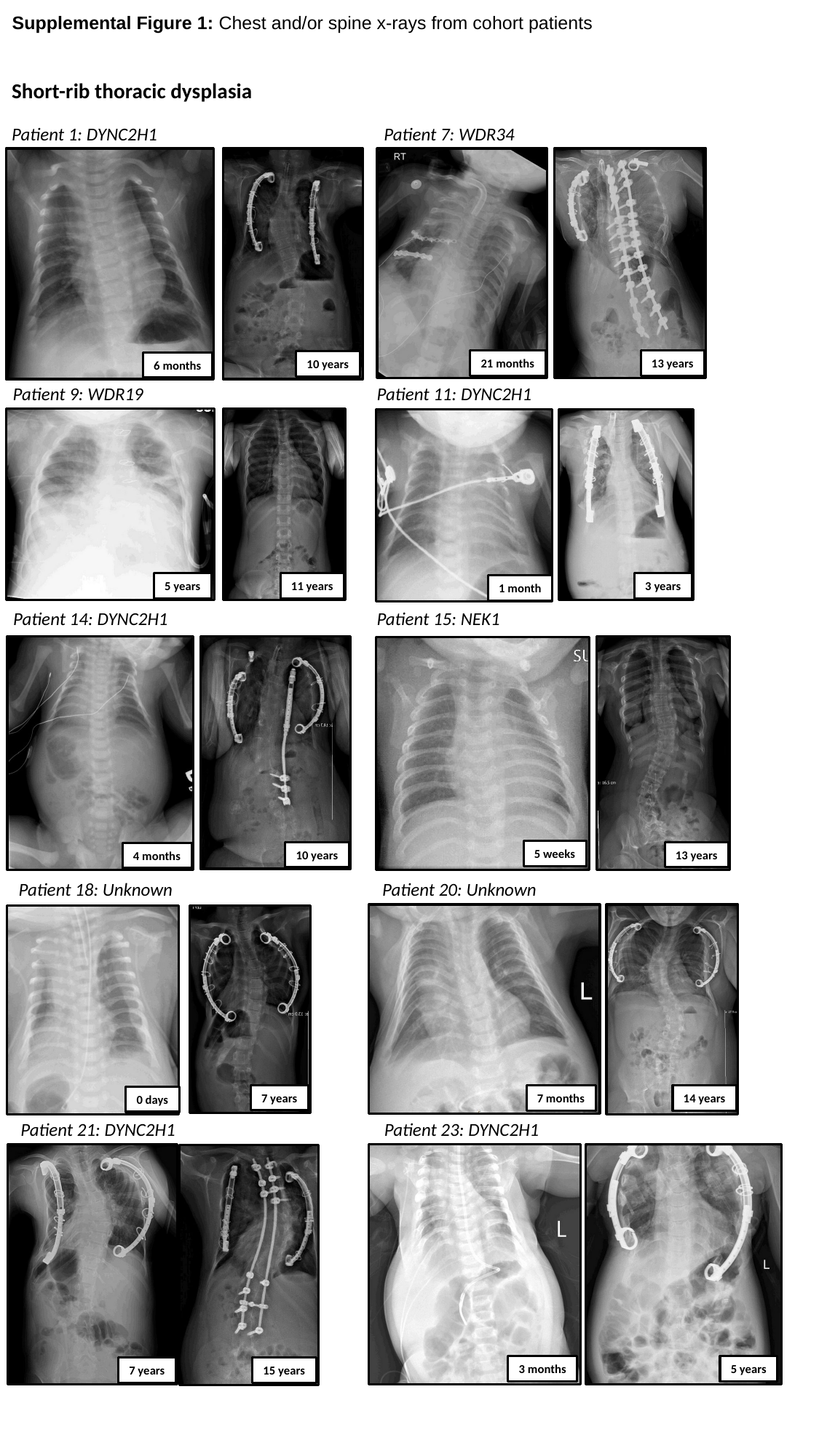

Supplemental Figure 1: Chest and/or spine x-rays from cohort patients
Short-rib thoracic dysplasia
Patient 1: DYNC2H1
Patient 7: WDR34
21 months
13 years
10 years
6 months
Patient 9: WDR19
Patient 11: DYNC2H1
5 years
11 years
3 years
1 month
Patient 14: DYNC2H1
Patient 15: NEK1
5 weeks
10 years
13 years
4 months
Patient 18: Unknown
Patient 20: Unknown
7 years
7 months
14 years
0 days
Patient 21: DYNC2H1
Patient 23: DYNC2H1
3 months
5 years
7 years
15 years

## Slide 2
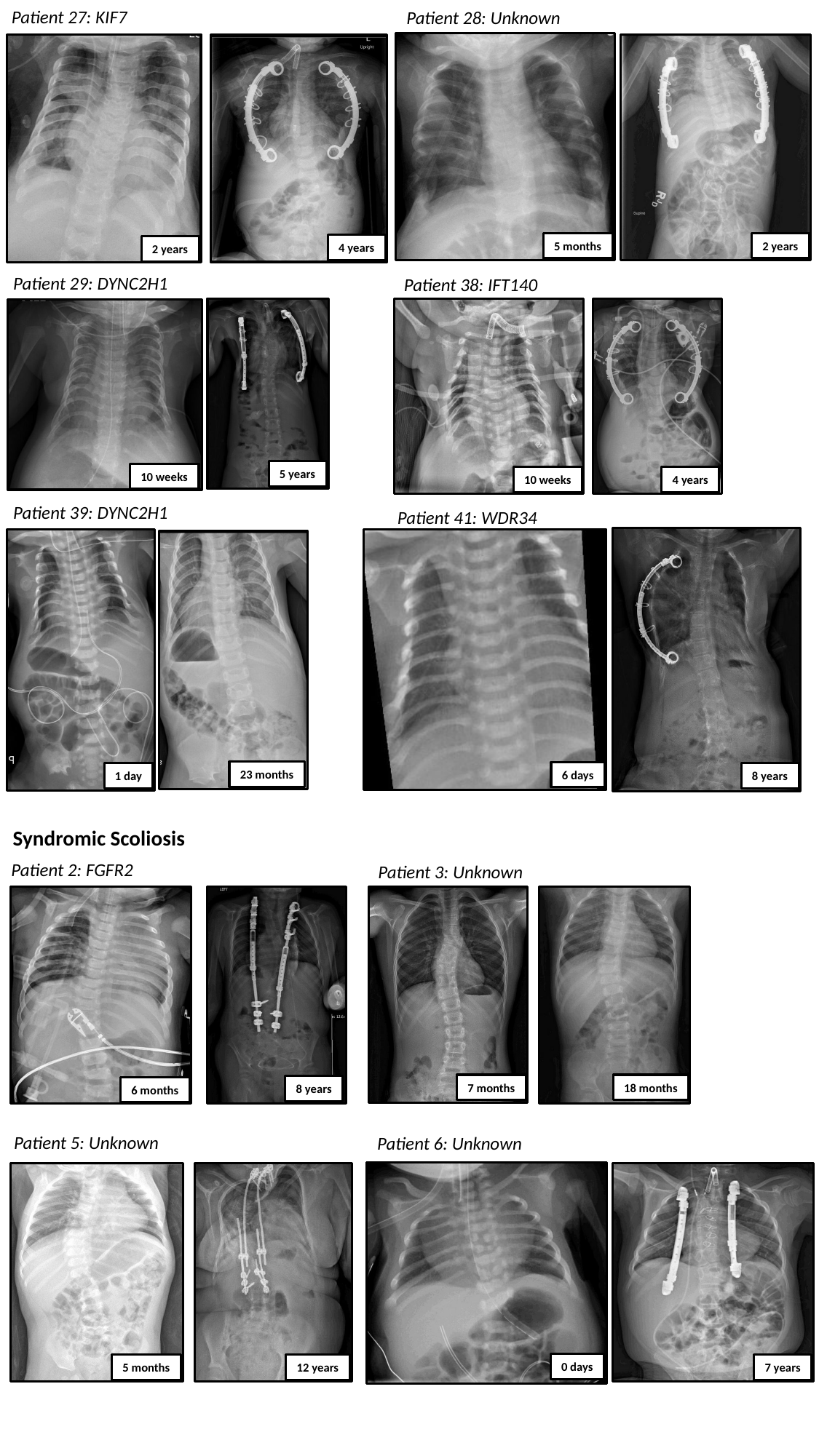

Patient 27: KIF7
Patient 28: Unknown
2 years
5 months
4 years
2 years
Patient 29: DYNC2H1
Patient 38: IFT140
5 years
10 weeks
4 years
10 weeks
Patient 39: DYNC2H1
Patient 41: WDR34
23 months
6 days
8 years
1 day
Syndromic Scoliosis
Patient 2: FGFR2
Patient 3: Unknown
7 months
18 months
8 years
6 months
Patient 5: Unknown
Patient 6: Unknown
0 days
5 months
12 years
7 years

## Slide 3
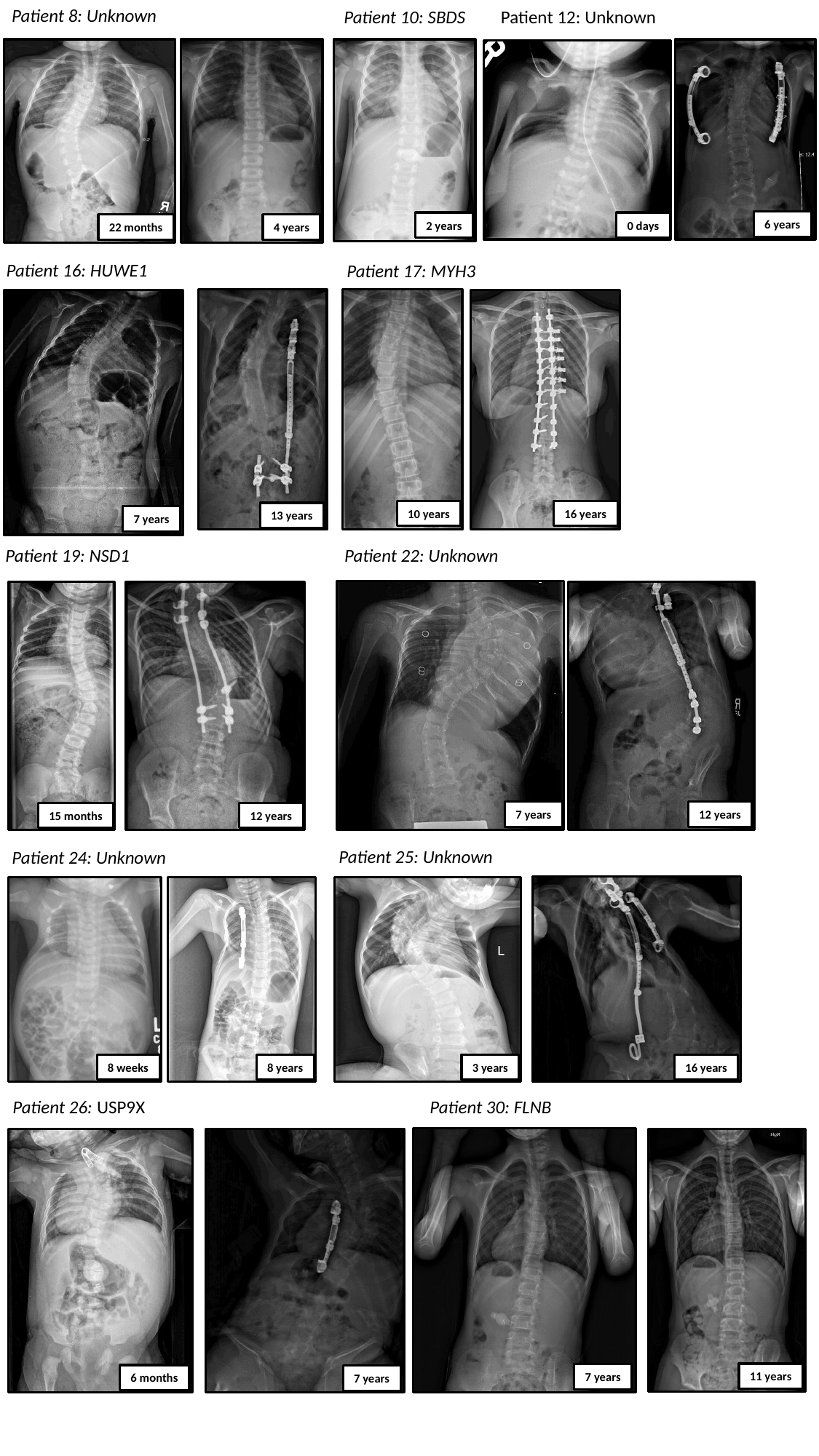

Patient 10: SBDS
Patient 12: Unknown
Patient 8: Unknown
6 years
2 years
0 days
22 months
4 years
Patient 16: HUWE1
Patient 17: MYH3
10 years
16 years
13 years
7 years
Patient 19: NSD1
Patient 22: Unknown
7 years
12 years
15 months
12 years
Patient 25: Unknown
Patient 24: Unknown
3 years
16 years
8 weeks
8 years
Patient 26: USP9X
Patient 30: FLNB
11 years
7 years
6 months
7 years

## Slide 4
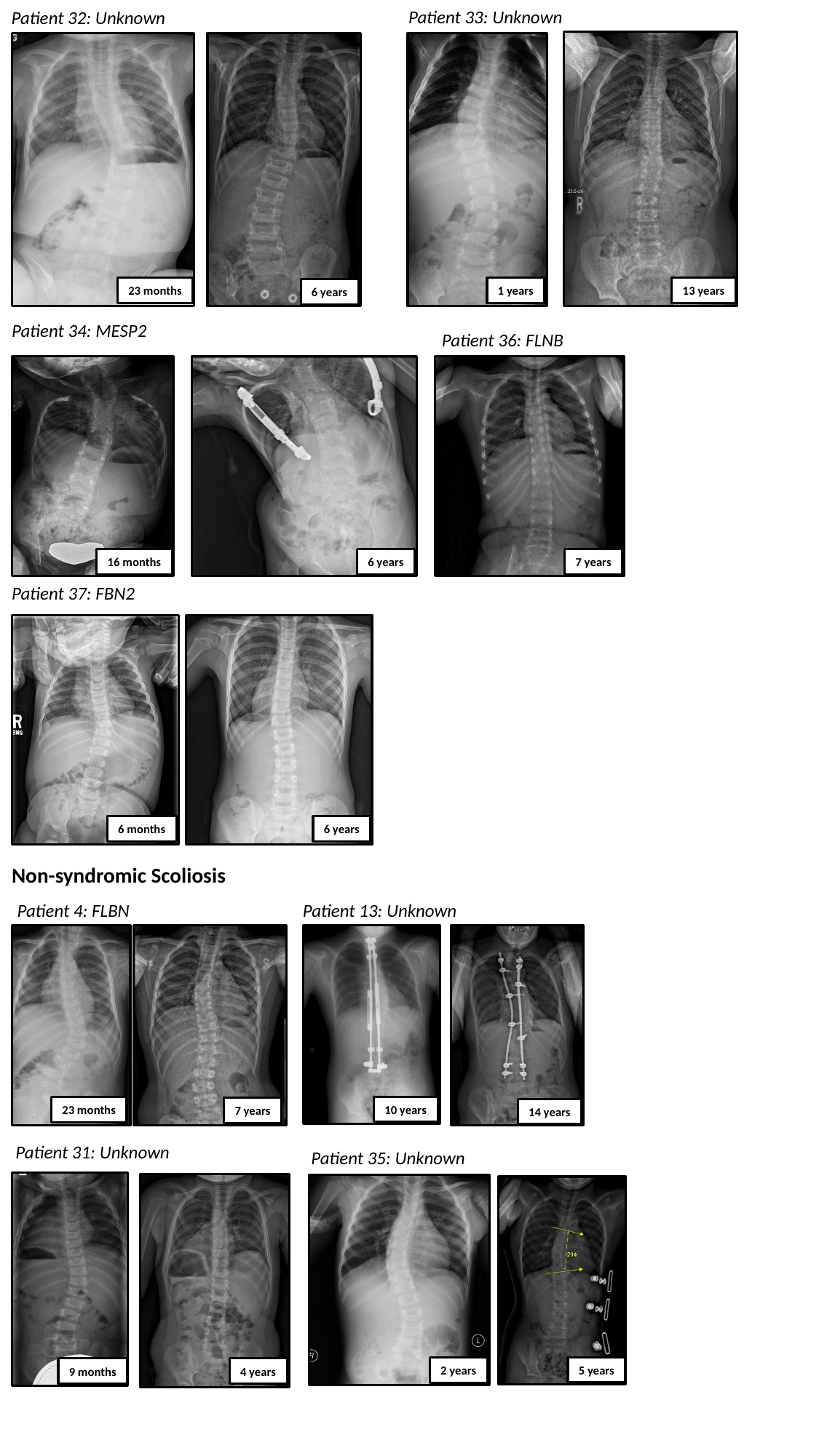

Patient 33: Unknown
Patient 32: Unknown
23 months
1 years
13 years
6 years
Patient 34: MESP2
Patient 36: FLNB
16 months
6 years
7 years
Patient 37: FBN2
6 months
6 years
Non-syndromic Scoliosis
Patient 4: FLBN
Patient 13: Unknown
10 years
23 months
7 years
14 years
Patient 31: Unknown
Patient 35: Unknown
5 years
2 years
9 months
4 years

## Slide 5
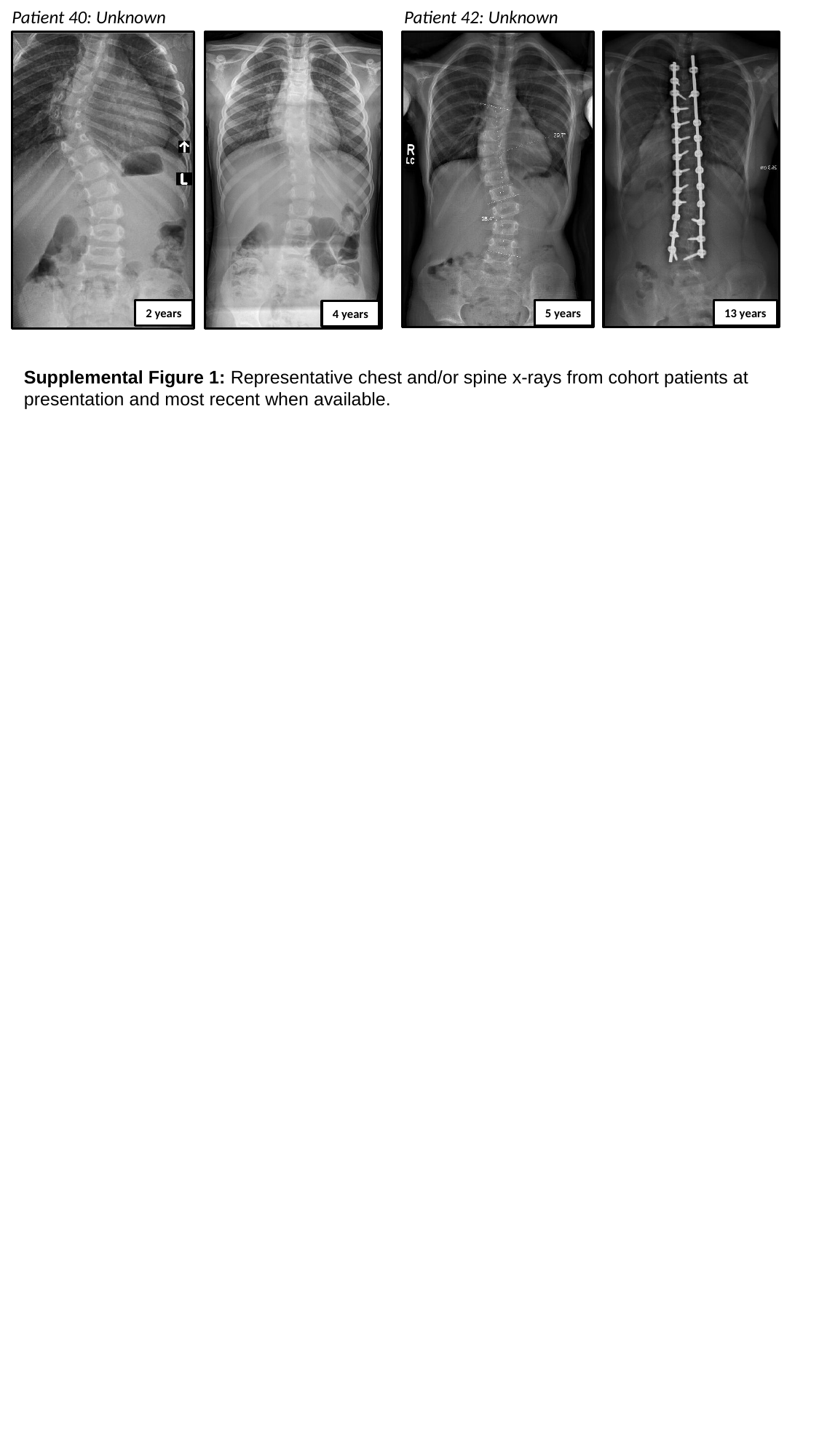

Patient 40: Unknown
Patient 42: Unknown
5 years
13 years
2 years
4 years
Supplemental Figure 1: Representative chest and/or spine x-rays from cohort patients at presentation and most recent when available.
